# A natural product YSK-A blocks SARS-CoV-2 propagation by targeting multiple host genes

**DOI:** 10.1038/s41598-023-48854-3

**Published:** 2023-12-06

**Authors:** Thuy X. Pham, Trang T. X. Huynh, Bumseok Kim, Yun-Sook Lim, Soon B. Hwang

**Affiliations:** 1 Laboratory of RNA Viral Diseases, Korea Zoonosis Research Institute, Jeonbuk National University, 820-120, Hana-Ro, Iksan, 54531 South Korea; 2 Laboratory of Veterinary Pathology, College of Veterinary Medicine, Jeonbuk National University, Iksan, South Korea; 3 Ilsong Institute of Life Science, Hallym University, Seoul, South Korea

**Keywords:** Microbiology, Diseases

## Abstract

Natural products and herbal medicine have been widely used in drug discovery for treating infectious diseases. Recent outbreak of COVID-19 requires various therapeutic strategies. Here, we used YSK-A, a mixture of three herbal components *Boswellia serrata*, *Commiphora myrrha*, and propolis, to evaluate potential antiviral activity against SARS-CoV-2. We showed that YSK-A inhibited SARS-CoV-2 propagation with an IC_50_ values of 12.5 µg/ml and 15.42 µg/ml in Vero E6 and Calu-3 cells, respectively. Using transcriptome analysis, we further demonstrated that YSK-A modulated various host gene expressions in Calu-3 cells. Among these, we selected 9 antiviral- or immune-related host genes for further study. By siRNA-mediated knockdown experiment, we verified that MUC5AC, LIF, CEACAM1, and GDF15 host genes were involved in antiviral activity of YSK-A. Therefore, silencing of these genes nullified YSK-A-mediated inhibition of SARS-CoV-2 propagation. These data indicate that YSK-A displays an anti-SARS-CoV-2 activity by targeting multiple antiviral genes. Although the exact antiviral mechanism of each constituent has not been verified yet, our data indicate that YSK-A has an immunomodulatory effect on SARS-CoV-2 and thus it may represent a novel natural product-derived therapeutic agent for treating COVID-19.

Natural products and herbal extracts have been used as traditional and complementary medicine, especially in Asian countries, including India, China, Japan, and Korea as well as in several African countries^1^. Since herbal medicine has an immunomodulatory effect, it could be used as a preventive measure and as a therapeutic agent for coronavirus disease 2019 (COVID-19) patients. Furthermore, natural products are less toxic, inexpensive, and easily accessible sources of phytochemicals for therapeutic agents. Therefore, complementary and substitute treatments using plant-based phytochemicals could be an alternative strategy for controlling severe acute respiratory syndrome coronavirus 2 (SARS-CoV-2) pandemic^2^. Indeed, National Health Commission of China has delivered therapy regulations for the use of herbal medicine as an alternative remedy for COVID-19 in combination with modern western medicine^3^. Accumulating evidence suggests that natural products derived from plants exert inhibitory effects on SARS-CoV-2 propagation. Herbal drug extracts from Polygala Root, Areca, and Quercus Bark and natural compounds derived from herbal drugs such as baicalin and glabridin display antiviral activity against SARS-CoV-2 infection^4^. In addition, PHELA, an herbal combination of four exotic African medicinal plants (*Clerodendrum glabrum E. Mey.* Lamiaceae*, Gladiolus dalenii* van Geel*, Rotheca myricoides* (Hochst.) Steane & Mabb, and *Senna occidentalis* (L.) Link), effectively inhibits both SARS-CoV and SARS-CoV-2 infections^5^.

The SARS-CoV-2 pandemic, a global outbreak of coronavirus, has had a devastating impact on the global economy and health of people across the world. To date, this virus has infected more than 770 million people worldwide and caused nearly 7 million deaths^6^. SARS-CoV-2 not only devastates the respiratory tract system^7^ but also affects other organs, and consequently leads to multiple organ failure and death^8,9^. Therefore, tremendous research efforts have been made to develop prevention and treatment strategies against SARS-CoV-2. So far, a few antiviral drugs (paxlovid, remdesivir, and molnupiravir) and more than 10 monoclonal antibodies have been marketed for COVID-19 treatment. However, the treatment window of antiviral agents is probably limited to the viral phase of SARS-CoV-2 infection. Apart from a few oral antivirals, current marketed antiviral agents are delivered by injection, further limiting practical administration in resource-limited settings lacking developed healthcare infrastructure^10^.

Natural products have been considered as sources of new anti-SARS-CoV-2 drugs. YSK-A is a mixture of three different herbal extracts consisting of *Boswelliaserrata, Commiphoramyrrha,* and propolis. Recently, we have reported that YSK-A exerts an antiviral activity by reducing lesions of respiratory tracts in SARS-CoV-2 infected hamsters^11^. To further investigate how YSK-A displays an anti-SARS-CoV-2 activity, we performed RNASeq analysis using Calu-3 cells and identified four host genes mainly involved in antiviral and immune responses. Importantly, we showed that YSK-A inhibited SARS-CoV-2 propagation by targeting MUC5AC, LIF, CEACAM1, and GDF15 genes. All these data suggest that YSK-A may represent a potential alternative natural product-derived antiviral agent against SARS-CoV-2. This is an important finding that natural products and herbal extracts could be utilized as resources for novel antiviral agents against SARS-CoV-2.

## Results

### YSK-A inhibits SARS-CoV-2 propagation

**Figure 1:**
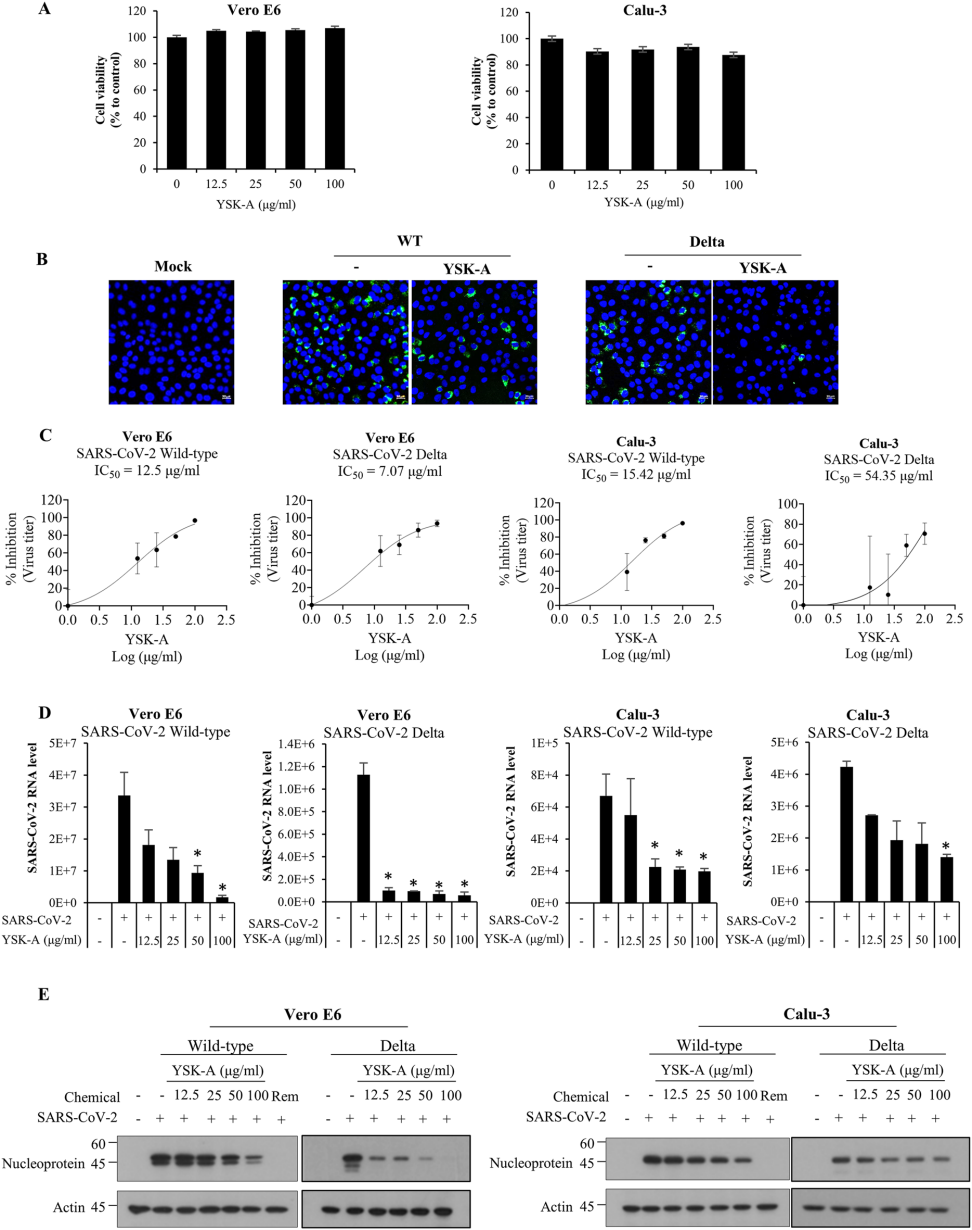
YSK-A impedes SARS-CoV-2 propagation. (**A**) Either Vero E6 or Calu-3 cells were treated with 12.5, 25, 50, 100 μg/ml of YSK-A. At 24 h (Vero E6) or 48 h (Calu-3) after treatment, cell viability was measured by WST assay as described in Material and Method. (**B**) Vero E6 cells were either mock-infected or infected with wild-type (MOI = 0.01) or Delta variant (MOI = 0.1) of SARS-CoV-2 for 1 h in the absence or presence of 100 μg/ml YSK-A. Cells were further cultured in media containing YSK-A. At 24 h postinfection, cells were fixed in 4% paraformaldehyde, and immunofluorescence staining was performed by using J2 antibody and FITC-conjugated goat anti-mouse IgG to detect double-stranded RNA (green). Cells were counterstained with DAPI to label nuclei (blue). Scale bar = 50 µm. (**C**–**E**) Cells were pretreated with 12.5, 25, 50, 100 μg/ml of YSK-A. At 24 h after treatment, cells were infected with wild-type (MOI = 0.01 in Vero E6; MOI = 0.1 in Calu-3) or Delta variant (MOI = 0.1 in Vero E6; MOI = 1 in Calu-3) of SARS-CoV-2 for 1 h in the absence or presence of the indicated concentrations of YSK-A. Cells were further cultured in media containing YSK-A and then harvested at 24 h postinfection for Vero E6 (**C**–**E**, left panels) and 48 h postinfection for Calu-3 (**C**–**E**, right panels). (**C**) Supernatant was collected to determine the infectious virus titer by TCID_50_ assay. For the “% inhibition” calculation, the normalized relative inhibition values were calculated according to the formula % inhibition = 100 x [1—(X—mock infected) / (infected untreated—mock infected)], where X is each given treatment condition. Dose–response curves for IC_50_ values were determined by nonlinear regression analysis using the GraphPad Prism v9 (GraphPad Software, San Diego, CA, USA). (D) SARS-CoV-2 RNA levels were determined by qRT-PCR. One-way ANOVA was used for comparing multiple YSK-A concentrations treatment with virus infection only. (**E**) Protein levels of SARS-CoV-2 were determined by an immunoblot assay using the indicated antibodies. Remdesivir (Rem) was used as a positive control. Data represent averages from triplicate experiments. *P < 0.05.

We have recently reported that YSK-A displayed potential antiviral activity against SARS-CoV-2^11^. To further evaluate whether YSK-A exerted anti-SARS-CoV-2 activity, we first evaluated cellular toxicity of YSK-A in Vero E6 and Calu-3 cells, which were known to be highly susceptible to SARS-CoV-2 infection^12^. We showed that as high as 100 µg/ml YSK-A displayed no cytotoxic effect on both cell lines (Fig. 1A). To determine whether YSK-A inhibits SARS-CoV-2 replication, Vero E6 cells were either mock infected or infected with either wild-type (MOI = 0.01) or Delta variant (MOI = 0.1) of SARS-CoV-2 and treated with either vehicle or 100 µg/ml YSK-A for 24 h and then SARS-CoV-2 replication was determined by staining cells with double-stranded RNA-specific J2 antibody. By using immunofluorescence assay, YSK-A profoundly decreased SARS-CoV-2 RNA in both wild-type and Delta variant (Fig. 1B). These data verified that YSK-A had anti-SARS-CoV-2 activity. To further confirm the anti-SARS-CoV-2 activity of YSK-A, either Vero E6 or Calu-3 cells were pretreated with various concentrations of YSK-A for 24 h and then infected with wild-type (MOI = 0.01 in Vero E6; MOI = 0.1 in Calu-3) or Delta variant (MOI = 0.1 in Vero E6; MOI = 1 in Calu-3) of SARS-CoV-2. At 24 h postinfection (Vero E6 cells) or 48 h postinfection (Calu-3 cells), supernatant was collected to determine the infectious virus titer by TCID_50_ assay. Simultaneously, RNA and protein levels of SARS-CoV-2 were analyzed either by quantitative real-time PCR (qRT-PCR) or immunoblot analysis. YSK-A significantly decreased infectious titer (Fig. 1C) and RNA levels (Fig. 1D) of SARS-CoV-2. We further showed that YSK-A markedly decreased protein levels of SARS-CoV-2 in a dose-dependent manner (Fig. 1E). Since remdesivir targets viral RNA-dependent RNA polymerase and widely used in SARS-CoV-2 research, we used remdesivir as a positive control. We demonstrated that 5 μM remdesivir completely abolished SARS-CoV-2 protein levels. Interestingly, we observed multiple bands of nucleoprotein (N) in Vero E6 cells. It has been previously reported that N of SARS-CoV undergoes posttranslational modifications^13^. The authors found both full-length N and additional bands, indicating the N-specific proteolytical cleavage with the help of Caspase-6. They further demonstrate that caspase-6-mediated cleavage of N depends on the cell lines and correlates with the replication cycle of SARS-CoV. N processing is only observed in Vero E6 and A549 cells but not in Caco-2 and N2a cells. We therefore postulate that SARS-CoV-2 N protein may also undergo similar posttranslational modifications. In fact, we previously observed multiple bands of N protein in Vero E6 cells^14^. Of note, YSK-A efficiently blocked virus titer with an IC_50_ value of 12.5 µg/ml in Vero E6 and 15.42 µg/ml in Calu-3 for wild-type, and IC_50_ value of 7.07 µg/ml in Vero E6 and 54.35 µg/ml in Calu-3 for Delta variant of SARS-CoV-2. Collectively, YSK-A displayed broad-spectrum antiviral activity, suggesting that it may represent a novel plant-derived therapeutic agent for COVID-19.

### YSK-A displays antiviral activity by regulating various host gene expressions

**Figure 2:**
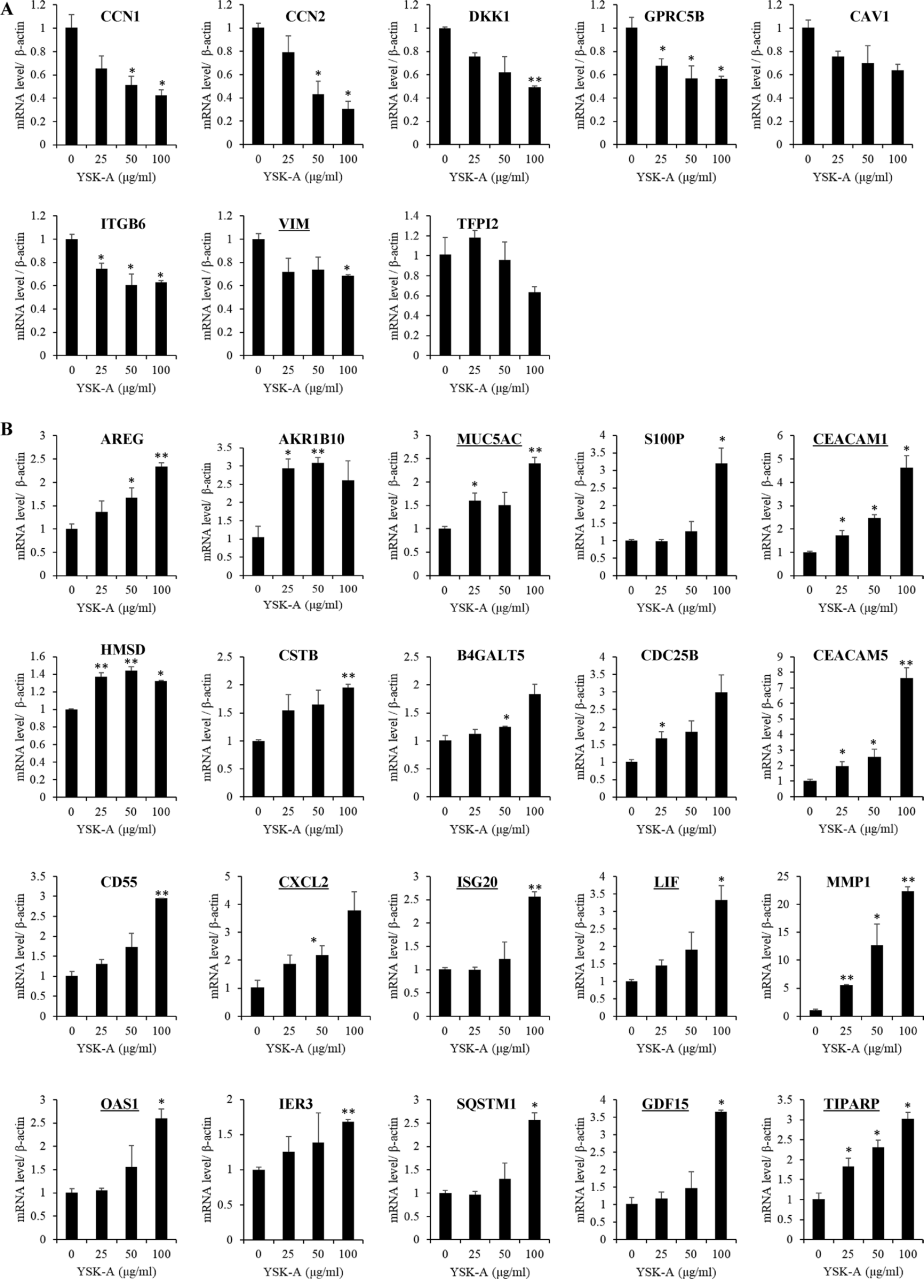
YSK-A modulates mRNA levels of numerous host genes. (**A**) Calu-3 cells were either mock treated or treated with 0, 25, 50, 100 μg/ml YSK-A. At 24 h after treatment, total cellular RNA was isolated and then cDNA was synthesized. mRNA levels of 8 host genes down-regulated by YSK-A were measured by qRT-PCR. (**B**) Calu-3 cells were treated as described in A and then mRNA levels of 20 host genes up-regulated by YSK-A were measured by qRT-PCR. Data represent averages from triplicate experiments. One-way ANOVA was used for comparing multiple YSK-A concentrations treatment with DMSO treatment (*P < 0.05; **P < 0.01).

To investigate the antiviral mechanism of YSK-A, we performed transcriptome sequencing (RNA-Seq) analysis using Calu-3 cells treated with 100 µg/ml YSK-A as we reported previously^15^. We identified ~ 100 host genes that were differentially expressed in YSK-A-treated cells. Of these, 39 genes were down-regulated with 1.5-fold reduction (Log_2_FC < -0.5) (Table 1A) and 65 genes were up-regulated with 1.5-fold increase (Log_2_FC > 0.5) in YSK-A treated cells (Table 1B). Of these differentially regulated host genes, we randomly selected 28 genes which are known to be involved in either antiviral activity or immune response. To further verify whether these selected genes were involved in YSK-A-mediated antiviral activity, Calu-3 cells were treated with increasing amounts of YSK-A. At 24 h posttreatment, RNA was isolated and then mRNA levels of these genes were measured by qRT-PCR. Figure 2A shows that mRNA levels of 8 genes were decreased by YSK-A in a dose dependent manner. Of note, YSK-A significantly decreased mRNA levels of CCN1, CCN2, DKK1, GPRC5B, ITGB6 and VIM with nearly twofold, which is consistent with transcriptomic data. Conversely, we further demonstrated that mRNA levels of 20 genes were increased by YSK-A in a dose dependent manner (Fig. 2B). The mRNA levels of 19 genes were similarly increased by YSK-A as seen in transcriptome data. However, the mRNA level of HMSD was increased only 1.5-fold as compared to sevenfold in transcriptomic analysis. This difference may be due to either the low copy number of HMSD in Calu-3 cells or the sensitivity level between these two methods. After literature search about these genes, we finally selected 1 down-regulated gene (VIM) and 8 up-regulated genes (MUC5AC, ISG20, LIF, OAS1, TIPARP, CEACAM1, CXCL2 and GDF15) to further investigate the potential roles of these in SARS-CoV-2 propagation.

**Table 1:** List of host target genes regulated by YSK-A.

ens_id	symbol	logFC	Pvalue	FDR
A				
ENSG00000288634	AC079741.2	-8.45	3.49E-13	7.75E-10
ENSG00000288661	AL451106.1	-7.06	1.80E-05	6.98E-03
ENSG00000288258	BX470111.1	-6.15	4.08E-03	6.80E-01
ENSG00000120738	EGR1	-2.23	1.10E-03	2.34E-01
ENSG00000205572	SERF1B	-2.04	4.20E-04	1.06E-01
ENSG00000118523	CCN2	-1.77	1.17E-09	1.56E-06
ENSG00000142871	CCN1	-1.70	1.25E-10	1.92E-07
ENSG00000105825	TFPI2	-1.24	3.89E-06	1.94E-03
ENSG00000107984	DKK1	-1.23	3.48E-08	3.36E-05
ENSG00000171223	JUNB	-1.23	1.25E-04	3.66E-02
ENSG00000019186	CYP24A1	-1.19	4.29E-17	1.71E-13
ENSG00000205413	SAMD9	-1.18	1.11E-03	2.34E-01
ENSG00000167191	GPRC5B	-1.13	7.80E-08	6.25E-05
ENSG00000115380	EFEMP1	-1.06	6.67E-03	9.53E-01
ENSG00000152952	PLOD2	-1.05	8.61E-05	2.73E-02
ENSG00000170525	PFKFB3	-1.05	1.13E-05	4.93E-03
ENSG00000134107	BHLHE40	-1.03	3.14E-03	5.61E-01
ENSG00000026025	VIM	-1.02	1.57E-11	2.85E-08
ENSG00000115221	ITGB6	-0.97	3.88E-04	1.00E-01
ENSG00000166920	C15orf48	-0.97	3.61E-05	1.34E-02
ENSG00000128335	APOL2	-0.93	9.79E-04	2.17E-01
ENSG00000100342	APOL1	-0.90	1.87E-06	1.10E-03
ENSG00000120708	TGFBI	-0.89	3.14E-09	3.92E-06
ENSG00000140416	TPM1	-0.88	5.71E-09	6.70E-06
ENSG00000128422	KRT17	-0.88	1.99E-06	1.11E-03
ENSG00000242265	PEG10	-0.83	4.69E-03	7.68E-01
ENSG00000162836	ACP6	-0.81	2.66E-03	4.88E-01
ENSG00000105974	CAV1	-0.81	7.50E-04	1.76E-01
ENSG00000166741	NNMT	-0.79	5.26E-03	8.28E-01
ENSG00000102024	PLS3	-0.70	9.03E-04	2.03E-01
ENSG00000137673	MMP7	-0.65	8.95E-05	2.79E-02
ENSG00000067167	TRAM1	-0.65	5.33E-03	8.29E-01
ENSG00000198959	TGM2	-0.65	1.54E-05	6.36E-03
ENSG00000167601	AXL	-0.64	6.20E-03	9.24E-01
ENSG00000135480	KRT7	-0.61	6.34E-08	5.50E-05
ENSG00000094755	GABRP	-0.56	6.60E-06	3.07E-03
ENSG00000117525	F3	-0.55	1.19E-03	2.48E-01
ENSG00000196924	FLNA	-0.51	9.26E-05	2.85E-02
ENSG00000117394	SLC2A1	-0.50	7.05E-04	1.70E-01

ens_id	symbol	logFC	PValue	FDR
B				
ENSG00000111321	LTBR	0.59	6.52E-03	9.53E-01
ENSG00000160213	CSTB	0.62	4.63E-07	3.35E-04
ENSG00000166986	MARS1	0.62	2.92E-04	7.87E-02
ENSG00000158470	B4GALT5	0.63	6.97E-03	9.87E-01
ENSG00000118849	RARRES1	0.66	1.31E-03	2.70E-01
ENSG00000167165	UGT1A6	0.68	9.62E-05	2.91E-02
ENSG00000160789	LMNA	0.69	2.00E-06	1.11E-03
ENSG00000053747	LAMA3	0.71	3.89E-06	1.94E-03
ENSG00000176153	GPX2	0.74	1.82E-05	6.98E-03
ENSG00000023330	ALAS1	0.74	1.02E-03	2.23E-01
ENSG00000183018	SPNS2	0.74	3.59E-03	6.19E-01
ENSG00000134827	TCN1	0.79	2.59E-05	9.77E-03
ENSG00000196139	AKR1C3	0.81	1.96E-08	2.06E-05
ENSG00000109321	AREG	0.84	2.37E-07	1.82E-04
ENSG00000115756	HPCAL1	0.86	2.56E-03	4.73E-01
ENSG00000161011	SQSTM1	0.87	4.04E-12	8.08E-09
ENSG00000198431	TXNRD1	0.87	4.70E-07	3.35E-04
ENSG00000197142	ACSL5	0.88	2.02E-03	3.91E-01
ENSG00000137331	IER3	0.89	5.07E-07	3.49E-04
ENSG00000139289	PHLDA1	0.90	2.26E-04	6.28E-02
ENSG00000124882	EREG	0.91	4.92E-10	7.02E-07
ENSG00000145335	SNCA	0.92	5.63E-03	8.64E-01
ENSG00000153292	ADGRF1	0.92	1.56E-05	6.36E-03
ENSG00000130066	SAT1	0.95	7.82E-08	6.25E-05
ENSG00000102804	TSC22D1	0.98	1.34E-03	2.73E-01
ENSG00000105963	ADAP1	1.01	1.61E-04	4.59E-02
ENSG00000136997	MYC	1.01	5.42E-06	2.64E-03
ENSG00000175592	FOSL1	1.01	1.11E-04	3.31E-02
ENSG00000131773	KHDRBS3	1.03	2.56E-03	4.73E-01
ENSG00000172183	ISG20	1.05	3.20E-03	5.61E-01
ENSG00000146674	IGFBP3	1.06	1.00E-10	1.67E-07
ENSG00000162337	LRP5	1.06	6.67E-03	9.53E-01
ENSG00000196352	CD55	1.09	1.06E-05	4.81E-03
ENSG00000198074	AKR1B10	1.09	5.86E-08	5.32E-05
ENSG00000174307	PHLDA3	1.10	4.84E-04	1.21E-01
ENSG00000215182	MUC5AC	1.14	7.26E-05	2.46E-02
ENSG00000187134	AKR1C1	1.19	5.68E-06	2.70E-03
ENSG00000113739	STC2	1.20	1.69E-04	4.75E-02
ENSG00000087842	PIR	1.21	2.52E-06	1.36E-03
ENSG00000148154	UGCG	1.21	1.11E-03	2.34E-01
ENSG00000101224	CDC25B	1.23	4.35E-05	1.58E-02
ENSG00000101187	SLCO4A1	1.27	1.59E-05	6.36E-03
ENSG00000151632	AKR1C2	1.27	1.29E-13	3.69E-10
ENSG00000103888	CEMIP	1.32	6.46E-04	1.57E-01
ENSG00000108846	ABCC3	1.37	9.20E-07	5.93E-04
ENSG00000128342	LIF	1.39	3.25E-13	7.75E-10
ENSG00000089127	OAS1	1.42	1.34E-06	8.34E-04
ENSG00000163993	S100P	1.42	1.11E-05	4.93E-03
ENSG00000081041	CXCL2	1.45	3.66E-03	6.24E-01
ENSG00000130513	GDF15	1.47	7.95E-04	1.85E-01
ENSG00000103044	HAS3	1.49	1.33E-05	5.64E-03
ENSG00000163659	TIPARP	1.50	3.53E-08	3.36E-05
ENSG00000184254	ALDH1A3	1.51	1.20E-26	1.19E-22
ENSG00000103089	FA2H	1.80	7.64E-17	2.54E-13
ENSG00000079385	CEACAM1	1.82	1.63E-06	9.89E-04
ENSG00000138678	GPAT3	1.91	4.32E-03	7.14E-01
ENSG00000180730	SHISA2	2.03	2.79E-06	1.47E-03
ENSG00000197632	SERPINB2	2.18	1.06E-08	1.18E-05
ENSG00000073737	DHRS9	2.45	4.88E-03	7.92E-01
ENSG00000221887	HMSD	2.74	7.89E-05	2.60E-02
ENSG00000105388	CEACAM5	2.76	6.37E-05	2.20E-02
ENSG00000114812	VIPR1	3.17	6.82E-07	4.54E-04
ENSG00000196611	MMP1	3.73	1.13E-93	2.25E-89
ENSG00000138061	CYP1B1	4.69	2.26E-22	1.50E-18
ENSG00000140465	CYP1A1	5.41	2.60E-18	1.30E-14

### YSK-A modulates mRNA levels of target genes at different time points

**Figure 3:**
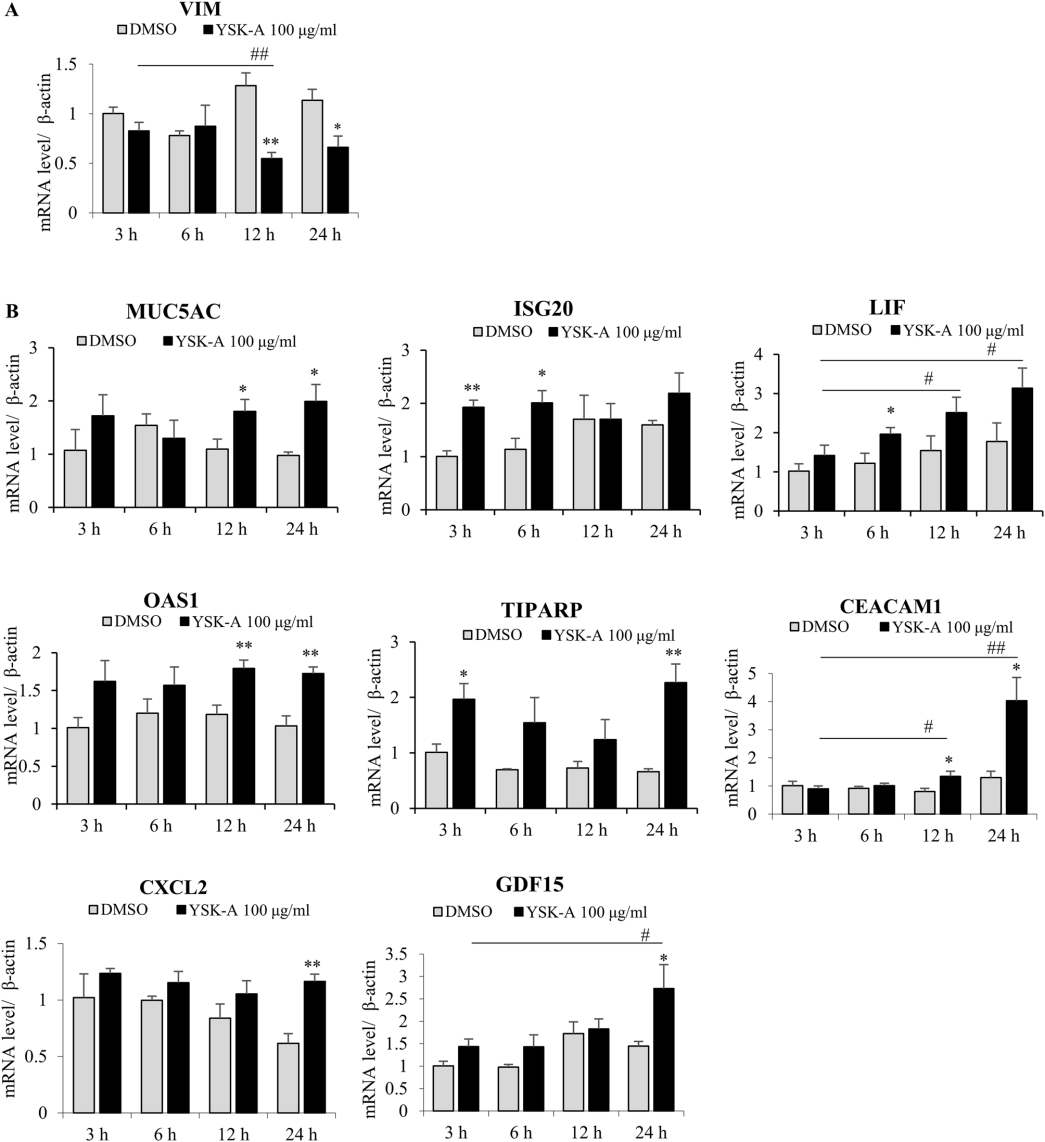
YSK-A regulates mRNA levels of target genes at various time points. (**A**) Calu-3 cells were either mock treated or treated with 100 µg/ml YSK-A. At the indicated time points, total cellular RNA was isolated and then cDNA was synthesized. mRNA level of VIM gene was measured by qRT-PCR. (**B**) Calu-3 cells were treated as described in A and then mRNA levels of 8 host genes were measured by qRT-PCR. Data represent averages from triplicate experiments. Student’s t test was used for comparing the differences between YSK-A treatments and mock-treatment at the indicated time points (*P < 0.05; **P < 0.01). One-way ANOVA was calculated to compare the differences between the indicated time points and mock-treatment at 3 h (#*P* < 0.05, ##*P* < 0.01).

To determine an optimum condition for treating cells with YSK-A, Calu-3 cells were treated with 100 µg/ml YSK-A. At the indicated time points after YSK-A treatment, RNA was extracted and then mRNA levels were analyzed by qRT-PCR. As shown in Fig. 3A, YSK-A significantly decreased mRNA level of VIM in Calu-3 cells at 12 h after treatment as compared with vehicle. Conversely, YSK-A significantly increased mRNA levels of other eight remaining genes (Fig. 3B). Interestingly, modulatory effects of YSK-A on target gene expressions varied at different time points. Therefore, mRNA levels were significantly upregulated at 12 h posttreatment in MUC5AC and OAS1, whereas at 3 h posttreatment in ISG20 and TIPARP. We further showed that YSK-A upregulated mRNA levels of LIF, CEACAM1, CXCL2, and GDF15 at 24 h posttreatment. For unknown reason, CXCL2 mRNA level in mock-treated cells was markedly decreased at 24 h posttreatment. Of note, modulatory effects of YSK-A on target genes were particularly significant at 24 h after treatment.

### Confirmation of the host target genes of YSK-A by siRNA-mediated silencing

**Figure 4:**
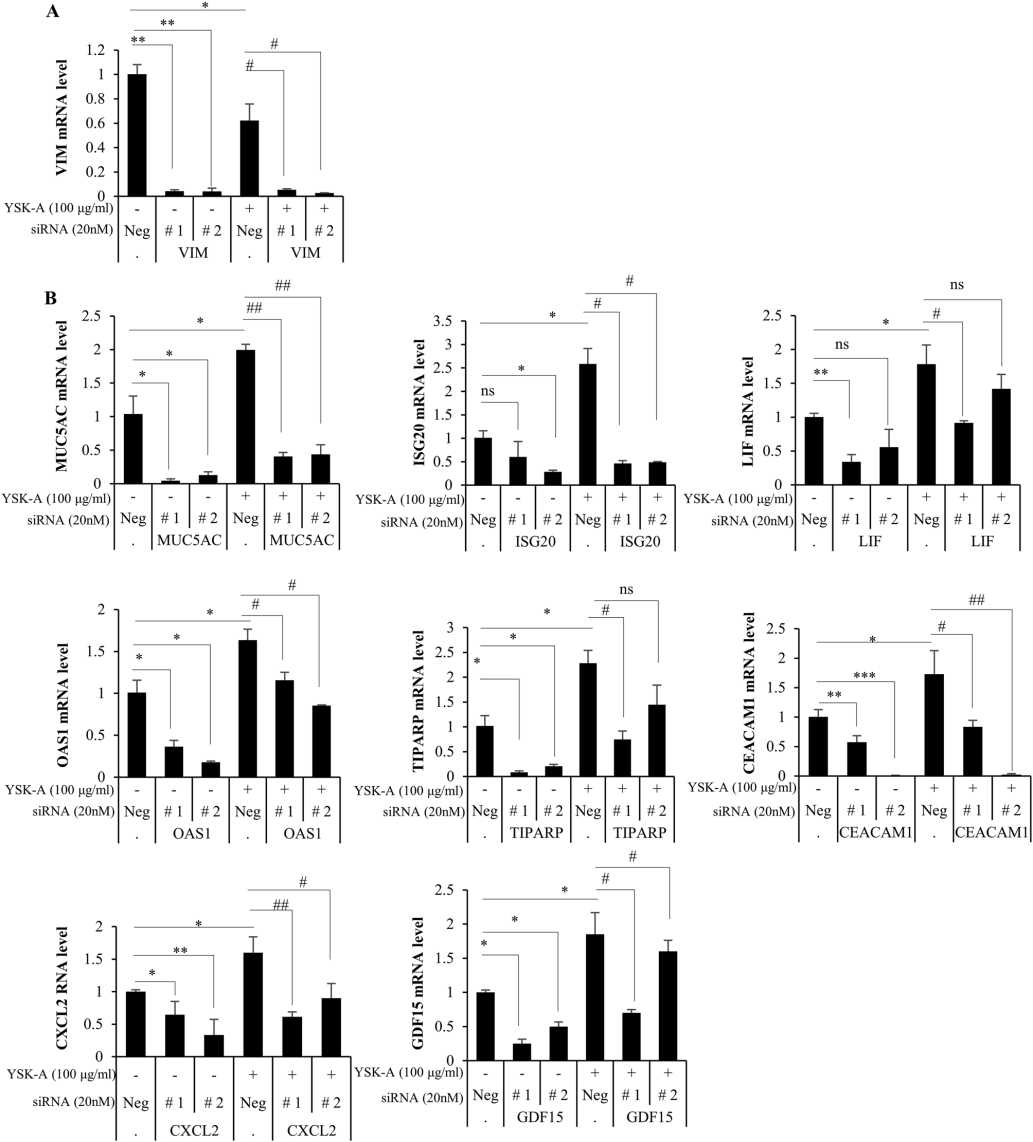
Confirmation of YSK-A target genes by siRNA-mediated silencing. (**A**) Calu-3 cells were transfected with either 20 nM negative siRNA or 20 nM VIM-specific siRNA. At 24 h after siRNA treatment, cells were treated with either DMSO or 100 µg/ml YSK-A. At 24 h after treatment, Calu-3 cells were harvested, RNA was extracted, and then mRNA expression level of VIM was determined by qRT-PCR. (**B**) Calu-3 cells were transfected with either 20 nM negative siRNA or 20 nM siRNAs targeting genes, including MUC5AC, ISG20, LIF, OAS1, TIPARP, CEACAM1, CXCL2 and GDF15. Cells were further treated as described in A, and then mRNA expression levels of 8 genes were determined by qRT-PCR. Data represent averages from triplicate experiments. One-way ANOVA was calculated to compare the difference between gene-specific siRNA and negative siRNA in the presence or absence of YSK-A. *P < 0.05; **P < 0.01, ***P < 0.001; ns, not significant; #*P* < 0.05; ##*P* < 0.01; *ns* not significant.

To further analyze the modulatory effect of YSK-A on selected host genes, Calu-3 cells were transfected with either negative siRNA or siRNA targeting each of 9 selected genes, followed by treatment with 100 µg/ml YSK-A. At 24 h after treatment, mRNA expression levels of YSK-A target genes were analyzed. As shown in Fig. 4, siRNA-mediated knockdown impaired mRNA expressions of all target genes. We further showed that YSK-A decreased mRNA levels of VIM nearly twofold (Fig. 4A), whereas YSK-A increased twofold in mRNA levels of MUC5AC, ISG20, LIF, OAS1, TIPARP, CEACAM1, CXCL2, and GDF15 (Fig. 4B). Therefore, modulatory role of YSK-A on mRNA expressions and knockdown efficiency of siRNAs were highly effective on target genes. Collectively, these data verified that siRNAs targeting nine genes properly decreased mRNA levels and these genes proved to be the real target of YSK-A.

### YSK-A displays anti-SARS-CoV-2 activity by targeting multiple host genes

**Figure 5:**
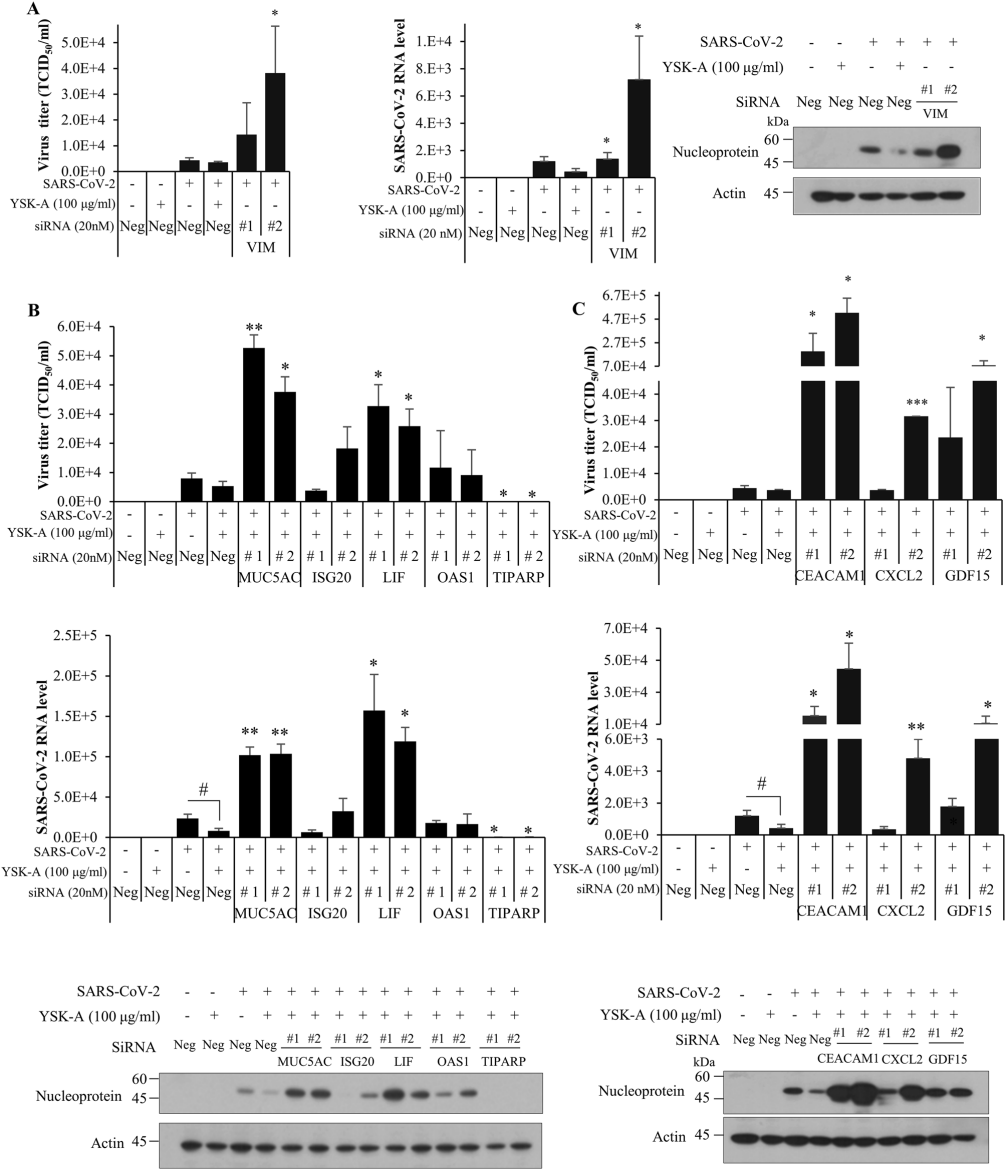
YSK-A displays anti-SARS-CoV-2 activity by targeting various host genes. (**A**) (Left panel) Calu-3 cells were transfected with either 20 nM negative siRNA or 20 nM VIM-specific siRNA. At 24 h after siRNA treatment, cells were treated with either DMSO or 100 µg/ml YSK-A. At 24 h after treatment, cells were either mock-infected or infected with wild-type SARS-CoV-2 (MOI = 0.2) in the absence or presence of 100 µg/ml YSK-A for 1 h. The cells were then washed with PBS and the culture medium was replaced with fresh medium containing either vehicle or 100 µg/ml YSK-A. At 48 h postinfection, TCID_50_ value was determined from the SARS-CoV-2-containing supernatant. (Middle panel) Total cellular RNA was extracted from the virus infected cells and then SARS-CoV-2 RNA levels were measured by qRT-PCR. (Right panel) Viral protein expression levels were determined by immunoblot assay using the indicated antibodies. (**B**) (Top panel) Calu-3 cells were transfected with either 20 nM negative siRNA or 20 nM siRNA targeting MUC5AC, ISG20, LIF, OAS1, and TIPARP, respectively. Cells were treated with YSK-A and further infected with SARS-CoV-2 as described in A. At 48 h postinfection, TCID_50_ value was determined from the SARS-CoV-2-containing supernatant. (Middle panel) Total cellular RNA was extracted from the virus infected cells and then SARS-CoV-2 RNA levels were measured by qRT-PCR. (Bottom panel) Viral protein expression levels were determined by immunoblot assay using the indicated antibodies. (**C**) (Top panel) Calu-3 cells were transfected with either 20 nM negative siRNA or 20 nM siRNA targeting CEACAM1, CXCL2, and GDF15, respectively. Cells were treated with YSK-A and further infected with SARS-CoV-2 as described in A. At 48 h postinfection, TCID_50_ value was determined from the SARS-CoV-2-containing supernatant. (Middle panel) Total cellular RNA was extracted from the virus infected cells and then SARS-CoV-2 RNA levels were measured by qRT-PCR. (Bottom panel) Viral protein expression levels were determined by immunoblot assay using the indicated antibodies. Data represent averages from triplicate experiments. Student’s t test was used for comparing the significant difference between gene-specific siRNA and negative siRNA in the presence of YSK-A (#*P* < 0.05). One-way ANOVA was calculated to compare the multiple treatments gene-specific siRNA and negative siRNA (*P < 0.05; **P < 0.01, ***P < 0.001).

Lastly, we interrogated whether YSK-A target genes are involved in SARS-CoV-2 propagation. For this purpose, Calu-3 cells were transfected with either negative siRNA or siRNA targeting the indicated host target genes. At 24 h after siRNA transfection, cells were further treated with either vehicle (DMSO) or 100 µg/ml YSK-A. One day after treatment, cells were infected with SARS-CoV-2 (MOI = 0.2) for 1 h in the absence or presence of YSK-A. At 48 h postinfection, SARS-CoV-2 propagation was analyzed by TCID_50_, qRT-PCR, and immunoblot analysis. As expected, YSK-A decreased virus titer, RNA, and nucleoprotein levels of SARS-CoV-2 as compared to DMSO treated cells. Since YSK-A downregulated the expression of VIM gene and VIM is considered to be a coreceptor of SARS-CoV-2^16,17^, we expected that knockdown of VIM might decrease SARS-CoV-2 propagation. However, silencing of VIM increased virus titer, RNA and protein levels of SARS-CoV-2 as compared to negative siRNA-treated cells (Fig. 5 A). These data suggest that VIM may not be involved in SARS-CoV-2 propagation. We next evaluated antiviral activities of the remaining 8 genes that were upregulated by YSK-A. We expected that if these gene expressions were impaired, YSK-A might no longer suppress the propagation of SARS-CoV-2. Indeed, YSK-A was unable to impede SARS-CoV-2 propagation in MUC5AC-, LIF-, CEACAM1- and GDF15-knockdown cells (Fig. 5B,C). Instead, knockdown of these four genes significantly increased viral propagation as compared to virus infection only. These data indicate that MUC5AC, LIF, CEACAM1, and GDF15 have intrinsic anti-SARS-CoV-2 activity and thus we propose that YSK-A may upregulate these four multiple genes to inhibit SARS-CoV-2 propagation. On the other hand, YSK-A marginally increased SARS-CoV-2 propagation in ISG20, OAS1, CXCL2 silencing cells. These data indicate that ISG20, OAS1, CXCL2 may not be involved in YSK-A-mediated SARS-CoV-2 antiviral activity. Of note, knockdown of TIPARP completely inhibited SARS-CoV-2 propagation in the presence of YSK-A, further suggesting that TIPRAP might not be involved in YSK-A-mediated SARS-CoV-2 regulation. Taken together, these data indicate that YSK-A inhibits SARS-CoV-2 propagation by upregulating antiviral- and immune-related multiple host target genes, including MUC5AC, LIF, CEACAM1, and GDF15.

## Discussion

Natural products and herbal medicine have been widely used to treat human diseases and disorders. We previously reported that *Mori Cortex Radicis*^18^, Triterpenoid saponins^19^, and saponin^20^ inhibit HCV propagation. It has been also reported that various natural products and their derivatives display antiviral activity against SARS-CoV-2^4,5,21^. We previously show that YSK-A exerts antiviral activity in SARS-CoV-2 infected cells^11^. In the current study, we showed that YSK-A displayed no cytotoxic effect on Vero E6 and Calu-3 cells. To further investigate how YSK-A displays an anti-SARS-CoV-2 activity, we performed RNASeq analysis and identified four host genes which have proved to be involved in antiviral and immune responses in YSK-A-treated Calu-3 cells. We demonstrated that YSK-A-regulated four host genes, including MUC5AC, LIF, CEACAM1 and GDF15, exhibited the antiviral activity against various variants of concern of SARS-CoV-2 in two different cell lines.

*Boswellia serrata* is known to have substantial anti-inflammatory activities^22–24^ and has been used to treat many inflammatory diseases such as rheumatoid arthritis^25^, ulcerative colitis^26^ and chronic colitis^27^. *Commiphora myrrha* has been demonstrated to have anti-inflammatory^28^ and analgesic activity^28,29^. It also has anti-microbia^30^ and anti-infective activity against acute respiratory viral infections^31^. In silico studies further suggest that *Boswellia serrata* ligands and a *Commiphora myrrha* product (Tiryaq-E-Wabai) may interact with viral proteins to interrupt SARS-CoV-2 propagation^32–34^. Propolis has been well known to have anti-microbial^35^, anti-inflammatory and immunomodulatory activities^36,37^ and it shows broad-spectrum antiviral activity against a diverse panel of viruses^38^. It has been previously reported that propolis exerts an antiviral effect on COVID-19^39,40^. In our previous study, *Commiphora myrrha* and propolis also displayed antiviral activity against SARS-CoV-2 in Vero E6 cells^11^.

To further investigate whether any of these host genes were involved in YSK-A-mediated antiviral activity, we performed RNA-Seq analysis in Calu-3 cells. We identified that ~ 100 host genes were differentially expressed in YSK-A-treated cells as compared with mock-infected cells (Table. 1). Of these, we selected 28 antiviral and immune-related genes, and investigated the effect of YSK-A on mRNA replication of target genes. Among these, we finally selected 1 down-regulated gene (VIM) and 8 up-regulated genes (MUC5AC, ISG20, LIF, OAS1, TIPARP, CEACAM1, CXCL2, GDF15) for further study. We showed that the modulatory effect of YSK-A on host genes has reached the maximum at 24 h after treatment. Based on this finding, we further analyzed antiviral activity of SARS-CoV-2 of MUC5AC, LIF, TIPARP, CEACAM1 and GDF15. Mucin 5AC (MUC-5AC) is a large gel-forming glycoprotein that forms a major airway mucin^41^. Recent study shows that SARS-CoV-2 spike induces MUC5AC/5B in human nasal epithelial cells^42^ and SARS-CoV-2 infection increases the MUC5AC secretion in primary human airway epithelial cells^43^. We showed that knockdown of MUC5AC nullified the inhibitory effect of YSK-A and thus enhanced SARS-CoV-2 infection. However, it has been previously reported that MUC5AC is riched in asthma^44^, and patients with asthma are vulnerable to get severe COVID-19^45^. Although mucus hyperproduction provides a physical barrier, IL-13 treated cells still maintain a low degree of infection despite the removal of mucus^43^. All these data suggest that MUC5AC acts as an antiviral factor and YSK-A regulates MUC5AC to inhibit SARS-CoV-2 propagation. Leukemia inhibitory factor (LIF) belongs to the IL-6 cytokine family. There is no direct evidence indicating that LIF exerts an anti-SARS-CoV-2 activity. It has been previously reported that LIF plays an important role in protecting lung from viral infection^46,47^. LIF is also required to maintain stable function of the blood-air barrier during infection^48^. In addition, our data showed that silencing of LIF increased SARS-CoV-2 propagation and thus LIF may be considered as an antiviral gene. The analysis of RNA-seq data from MERS-CoV infected Calu-3 cells detected the up-regulation of AHR and related genes including TCDD-inducible poly-ADP-ribose polymerase (TIPARP)^49^**.** TIPARP is a viral RNA-sensing pattern recognition receptor that mediates antiviral responses triggered by BAX- and BAK1-dependent mitochondrial damage^50^. However, TIPARP inhibits IFN production by ADP-ribosylation of TBK-1, leading to enhanced replication of several viruses^51^. In our study, YSK-A virtually abolished SARS-CoV-2 propagation in TIPARP-silenced cells. This result suggests that other mechanism may be involved in the YSK-A-regulated TIPARP function of the SARS-CoV-2 propagation. Carcinoembryonic antigen-related cell adhesion molecule 1 (CEACAM1**)** is involved in IFN-γ-mediated induction of inflammatory responses, cellular growth, and proliferation in airway epithelial cells^52^. CEACAM1 expression level is the highest during SARS-CoV-2 infection, followed by SARS-CoV, IAV, and RSV^53^. Moreover, CEACAM1 suppresses both HCMV and influenza viruses in an SHP2-dependent process by suppressing mTOR-mediated protein biosynthesis^54^. Overall, CEACAM1 functions as an antiviral factor and YSK-A may exert an anti-SARS-CoV-2 activity by increasing CEACAM1 expression level. Growth and differentiation factor 15** (**GDF15**)** belongs to the transforming growth factor β superfamily and is also named macrophage inhibitory cytokine-1. Overexpression of GDF15 inhibits H5N1 infection^55^. GDF15 is known to be a biomarker of cardiovascular and inflammatory COVID-19 patients in many clinical studies^56^. Overexpression of GDF15 in COVID-19 might be a compensatory mechanism to counteract dysregulated inflammatory reactions^57^. Here we postulate that YSK-A may display an antiviral activity by modulating GDF15 expression level in SARS-CoV-2 infection settings. However, overexpression of GDF15 increases the infectivity of HCV^58^. Since the role of GDF15 is still controversial in various viral infections, more studies are needed to elucidate the molecular mechanism of GDF15 in YSK-A-treated SARS-CoV-2 infection.

To further determine which host genes might be involved in YSK-A-mediated antiviral activity, we also selected interferon stimulated exonuclease gene 20 (ISG20), 2′,5′-oligoadenylate synthetase 1 (OAS1), and C-X-C motif chemokine ligand 2 (CXCL2) because these genes are known to have intrinsic antiviral activity. ISG20 has been shown to inhibit multiple viruses, including hepatitis A virus (HAV), hepatitis B virus (HBV), hepatitis C virus (HCV), Influenza A virus (IAV), and SARS-CoV-2^59–63^. Thus, targeting ISG20 may be a potential therapeutic strategy for treating COVID-19. OAS1 is an interferon-stimulated gene that plays a key role in the cellular innate immune response. A recent study shows that OAS1 binds to dsRNA structures in the SARS-CoV-2 5′-UTR and blocks SARS-CoV-2 replication^64^. Using inducible CRISPR activation screen, OAS1 is identified as a SARS-CoV-2 restriction factor^65^ and decreased OAS1 expression contributes to COVID-19 severity^66^. CXCL2 is involved in inflammatory response. CXCL2 can clear SARS-CoV in mice depleted with CD4 + cells^67^. Although ISG20, OAS1 and CXCL2 have intrinsic antiviral activity, these genes are not involved in inhibitory activity of YSK-A in SARS-CoV-2 propagation. VIM, a type III intermediate filament protein, has been considered as coreceptors for various viruses^68^. It has been reported that VIM plays a crucial role in SARS-CoV infection^69^. VIM may bind to SARS-CoV-2 S protein and facilitate SARS-CoV-2 entry in human endothelial cells^16,17^. However, our study showed that silencing of VIM increased SARS-CoV-2 propagation. Further study is needed to determine whether VIM is involved in YSK-A-regulated SARS-CoV-2 propagation.

Collectively, we demonstrated that YSK-A inhibited SARS-CoV-2 propagation through multiple host genes which have widely been involved in antiviral activity and immune response. The mode of inhibitory action of YSK-A in SARS-CoV-2 propagation may be a complicated process because YSK-A consists of 3 different natural products which exhibit their own antiviral activity and immunomodulatory response. Nevertheless, using SARS-CoV-2-infected cells as a model system, we verified that YSK-A blocked viral propagation by modulating antiviral and immune-related host genes. Most importantly, we provided crucial evidence that natural products derived from plants could be an alternative therapeutic option for the treatment of COVID-19. In conclusion, YSK-A displays an anti-SARS-CoV-2 activity and thus potential of our findings are profound. However, further clinical studies are necessary to evaluate the efficacy of YSK-A as therapeutic agents for COVID-19.

## Methods

### Cell culture

Vero E6 cells derived from the kidney of an African green monkey and Calu-3 cells derived from human lung cancer were grown in Dulbecco’s modified Eagle’s medium (DMEM) supplemented with 10% fetal bovine serum, 1% penicillin–streptomycin, and 1% nonessential amino acids with 5% CO_2_ at 37 °C. Cells were used in experiments from 3 to 10 passages and viral infection was performed when the cell density was ~ 90%. Calu-3 cells were provided by Korean Cell Line Bank (Seoul, Korea) and Vero E6 cells were obtained from ATCC.

### Preparation of infectious SARS-CoV-2

Wild-type Wuhan (NCCP-43331) and Delta (NCCP-43405) of SARS-CoV-2 were provided by the National Culture Collection for Pathogens, South Korea. Viruses were cultured in Vero E6 cells grown in DMEM supplemented with 2% FBS, 1% penicillin–streptomycin, and HEPES (Invitrogen, USA). Viral titers were determined by the 50% tissue culture infectious dose (TCID_50_) assay. All experiments were conducted in a biosafety level 3 (BSL-3) facility, the Korea Zoonosis Research Institute, Jeonbuk National University.

### YSK-A preparation

A powder mixture (1:1:1) of *Boswellia serrata*, *Commiphora myrrha*, and propolis (designated as YSK-A) used in this study was provided by Yeskin (Korea). *Boswellia serrata* ethyl alcohol (EtOH) extract powder (UMALAXM ORGANICS Pvt. Ltd, June 2020) and propolis extract powder (AC biotech Pty. Ltd, October 2020) are commercially available products. *Commiphora myrrha* extract powder was obtained as follows. *Commiphora myrrha* (SAMIN pharma. INC., December 2021) was extracted with 50% EtOH at 90 °C for 8 h. The extract was concentrated and prepared as a freeze-dried powder. Standard compounds cinnamic acid, galangin, 11-keto-β boswellic acid (KBA), acetyl-11-keto-β-boswellic acid (AKBA), and furanoeudesma 1,3-diene were purchased from Coresciences. Then the YSK-A quantity was tested and the purity of all components were more than 95%^11^.

### Water-soluble tetrazolium salt (WST) assay

Vero E6 or Calu-3 cells seeded on a 24-well plate were treated with various concentrations of YSK-A. At the indicated time points, cell viability was measured using 30 µl of WST (Dail Lab, Korea) as reported previously^70^.

### TCID_50_ assay

A 50% tissue culture infectious dose (TCID_50_) assay was performed to determine the infectious titer of cell culture-produced SARS-CoV-2 as described previously^71^ with few modifications. Vero E6 cells seeded on 96-well plates overnight were infected with ten-fold serial dilutions of the virus-containing supernatants. At 5 days postinfection, virus-infected cells were counted by the presence of CPE in each well under a light microscope.

### Transcriptome analysis

#### RNA purification

Calu-3 cells were treated with 100 µg/ml YSK-A. At 24 h post-treatment, cells were harvest and then total RNA was extracted using NucleoZol (Macherey–Nagel, Germany) according to the manufacturer's method.

#### RNA sequencing library construction

For the RNA sequencing assay, cDNA libraries were constructed, and single-end libraries were sequenced using the MGI-T7 platform (MGI Tech Co) using MGIEasy RNA Directional Library Prep Set.

#### Sequence alignment

We removed adapter sequence using Cutadapt version 2.9 and using the quality filtering of Trimmomatic version 0.39 which scans through reads from the 5’end and removes reads that length under 36 bases. After Trimming, reads were aligned to the human reference genome consisting of hg38 and Ensembl v.102 used STAR version 2.7.3a. RSEM version 1.3.1. was used in combination with STAR program. STAR and RSEM were set default parameters^72–75^.

#### Gene expression analysis

We normalized gene count data used DEseq2 normalized method into coding genes. We performed gene expression pattern to heatmap and PCA plot using R packages. Differential expression of gene analysis was performed using DEseq2^76^.

#### Difference expression of genes

We used DESeq2 for DEG identification from RNA-seq data that normalized expect counts at fold change > 1.5. We then classified the gene expression into 2 groups: down-regulated group with 1.5-fold reduction (Log_2_FC <  − 0.5) and up-regulated group with 1.5-fold increase (Log_2_FC > 0.5).

### Quantification of RNA

Total RNA was isolated using NucleoZol (Macherey–Nagel, Germany). cDNA was synthesized by using a cDNA synthesis kit (Toyobo) according to the manufacturer’s instructions. Quantitative real-time PCR (qRT-PCR) experiments were performed using the CFX Connect real-time system (Bio-Rad Laboratories, Hercules, CA). Primer sequences to detect YSK-A-stimulated genes, β-actin and SARS-CoV-2 polymerase are listed in Table 2.

**Table 2:** Primer sequences.

Primer	Primer sequence sense (5'-3')
LIF – F	GGCATAGGAACAATCTGGCAGA
LIF—R	TCCTCCTCCCAGTGTCTTCC
TIPARP – F	ACCACCCTCTAGCAATGTCAA
TIPARP—R	CCGAGAGTTGGCTTCTTCAATC
CEACAM1 – F	CTGCACAGTACTCCTGGCTT
CEACAM1—R	TTGCAGCCAGTGACTGAGTT
CXCL2 – F	TCTTCGTGATGACATATCACATGTC
CXCL2—R	ACACATACATTTCCCTGCCGTC
RdRp – F	GTG AAA TGG TCA TGT GTG GCG G
RdRp—R	CAA ATG TTA AAA ACA CTA TTA GCA TA
β-actin—F	TGA CAG CAG TCG GTT GGA GCG
β-actin—R	GAC TTC CTG TAA CAA CGC ATC TCA TA
Primers for other targets were purchased from Bioneer, South Korea	

### RNA interference

siRNAs targeting YSK-A-stimulated genes and the universal negative control siRNA were purchased from Bioneer (South Korea). Target sequences for siRNAs are listed in Table 3. siRNA transfection was performed using a Lipofectamine RNAiMax reagent (Invitrogen, Carlsbad, CA) according to the manufacturer’s instructions.

**Table 3:** Target sequences for siRNAs.

siRNA	RNA sequence sense (5'-3')
MUC5AC #1	CAC AAC CTC TGC TCC TAC A
MUC5AC #2	CAC AAC CTT AGC TCC TAC A
ISG20 #1	GGA CAG CAA CTA CCT TGC T
ISG20 #2	CTA AGT AGC ATG TGG CTT A
LIF #1	GAA CAA TCT GGC AGA AGT T
LIF #2	CAG ATG TTC CTG CCT TAG A
OAS1 #1	GTC AAG CTC ATC GAG GAG T
OAS1 #2	CAG AAA GAG GGC GAG TTC T
TIPARP #1	GAG AGT ATC CCG AGT CTG
TIPARP #2	CTG TGT TGT TTA AGC AAG A
VIM #1	TGA AGC TGC TAA CTA CCA A
VIM #2	CTA ACT ACC AAG ACA CTA T
CEACAM1 #1	GAG CTA GAT TTA ACT CAG T
CEACAM1 #2	CAG TAC TCC TGG CTT ATC A
CXCL2 #1	GAT AGA AGG TTT GCA GAT A
CXCL2 #2	TCT TCG TGA TGA CAT ATC A
GDF15 #1	CAG AGT TGC ACT CCG AAG A
GDF15 #2	CTC AGA GTT GCA CTC CGA A

### Immunoblot analysis

Cells were harvested and lysed in a buffer containing 50 mM Tris HCl (pH 7.5), 150 mM NaCl, 1% NP-40, 1 mM EDTA, 0.25% sodium deoxycholate, 1 mM Na_3_VO_4_, 1 mM sodium fluoride, 1 mM phenylmethylsulfonyl fluoride, 1 mM β-glycerophosphate, and protease inhibitor cocktail (Roche) for 30 min on ice, and centrifuged at 12,000 rpm for 10 min at 4 °C. The supernatant was collected and equal amounts of protein were subjected to SDS-PAGE and then electrotransferred to a nitrocellulose membrane. The membrane was blocked in TBST buffer (20 mM Tris–HCl (pH 7.6), 150 mM NaCl, and 0.2% Tween 20) containing 5% non-fat dry milk for 1 h and then incubated overnight at 4 °C with the indicated antibodies in TBST buffer containing 1% BSA. The SARS-CoV-2 nucleoprotein was detected using a primary anti-nucleoprotein antibody (1/4000 dilution) (Sino Biological, China), and the secondary goat anti-rabbit antibody (1/5000 dilution) (Jackson ImmunoResearch Laboratories, West Grove, PA, USA). Actin was detected by the monoclonal anti-actin primary antibody (1/3000 dilution) (Sigma- Alrdrich, USA) and the secondary goat anti-mouse antibody (1/5000 dilution) (Jackson ImmunoResearch Laboratories, West Grove, PA, USA). Proteins were detected using an ECL kit (Amersham Biosciences).

### Immunofluorescence assay

Vero E6 cells seeded on cover glass were infected with SARS-CoV-2 at an MOI of 0.01 in the absence or presence of 100 µg/ml YSK-A for 24 h. Cells were rinsed in PBS and fixed with 4% paraformaldehyde for 10 min. After two washes in PBS, fixed cells were permeabilized with 0.1% Triton X-100 in PBS for 15 min. Cells were then blocked in 0.5% BSA in PBS for 1 h and incubated overnight with mouse monoclonal anti-dsRNA J2 antibody (1/1000 dilution) (English & Scientific Consulting, J21003). After three washes in PBS, cells were incubated with FITC-conjugated goat anti-mouse (1/1000 dilution) (Jackson ImmunoResearch Laboratories, West Grove, PA, USA). Cells were also counterstained with 4′,6′-diamidino-2-phenylindole (DAPI) for 10 min to label nuclei. Fluorescence was analyzed by using the Zeiss LSM 700 laser 397 confocal microscopy system (Carl Zeiss, Inc., Thornwood, NY) and CELENA® S Digital Imaging System 398 (Logos Biosystems, Inc.).

### Statistical analysis

Data are presented as the means ± standard deviations (SDs). Statistical analysis was performed by Student’s *t* test for two treatments and one-way analysis of variance (ANOVA) for multiple treatments. Dunnett’s test was used as a post hoc test. All statistical analyses were performed using IBM® SPSS® statistic v22 (IBM). Half-maximal effective (IC_50_) concentrations of YSK-A were estimated by nonlinear regression analysis using the GraphPad Prism v9 (GraphPad Software, San Diego, CA, USA). All graphs were drawn using GraphPad Prism and Excel 2016 (Microsoft). The asterisks or sharp in the figures indicate significant differences (*P < 0.05; **P < 0.01; ***P < 0.001; ns, not significant).

## Data Availability

The datasets generated and analyzed during the current study are available from the corresponding author upon reasonable request.

## Abbreviations

COVID-19: Coronavirus disease 2019
SARS-CoV-2: Severe acute respiratory syndrome coronavirus 2
